# Oral administration of the amino acids cystine and theanine attenuates the adverse events of S-1 adjuvant chemotherapy in gastrointestinal cancer patients

**DOI:** 10.1007/s10147-016-0996-7

**Published:** 2016-06-15

**Authors:** Takashi Tsuchiya, Hiroshi Honda, Masaya Oikawa, Tetsuya Kakita, Atsushi Oyama, Hidekazu Oishi, Katsuyuki Tochikubo, Takanao Hashimoto, Shigekazu Kurihara, Tetsuro Shibakusa, Takashi Kayahara

**Affiliations:** 1 Department of Surgery, Sendai City Medical Center, 5-22-1 Tsurugaya, Miyagino-ku, Sendai, Miyagi 983-0824 Japan; 2 Department of Pharmacy, Sendai City Medical Center, Sendai, Japan; 3 Institute for Innovation, Ajinomoto Co., Inc., Kanagawa, Japan; 4 Wellness Business Division, Ajinomoto Co., Inc., Tokyo, Japan

**Keywords:** Adverse events, Chemotherapy, Cystine/theanine, S-1, Supportive care

## Abstract

**Background:**

Nutritional therapy is used to reduce the adverse events (AEs) of anticancer drugs. Here, we determined whether the amino acids cystine and theanine, which provide substrates for glutathione, attenuated the AEs of S-1 adjuvant chemotherapy.

**Methods:**

Patients scheduled to receive S-1 adjuvant chemotherapy were randomized to the C/T or the control groups. The C/T group received 700 mg cystine and 280 mg theanine orally 1 week before the administration of S-1, which then continued for 5 weeks. Each group received S-1 for 4 weeks. Blood sampling was performed and AEs were evaluated (CTCAE ver. 4.0) before and after the administration of S-1. S-1 was discontinued when AEs ≥ grade 2 occurred.

**Results:**

The incidences of AEs of any grade and those over grade 2 were lower in the C/T group than in the controls. The incidence of diarrhea (G ≥ 2) was significantly less (*p* < 0.05) in the C/T group (3.1 %) than in the controls (25.8 %). The duration and completion rate of the S-1 adjuvant chemotherapy were significantly longer (*p* < 0.01) and higher (*p* < 0.01), respectively, in the C/T group (complete ratio: 75.0 %, duration: 24.8 ± 5.8 days) than in the controls (complete ratio: 35.5 %, duration: 20.0 ± 7.7 days).

**Conclusions:**

The oral administration of cystine and theanine attenuated the AEs of S-1 adjuvant chemotherapy and increased the S-1 completion rate, suggesting that cystine and theanine is a useful supportive care for chemotherapy.

## Introduction

Adherence to the dosing schedule is important for the effectiveness of anticancer chemotherapy and affects therapeutic outcomes [1]. To improve adherence, measures that reduce adverse events (AEs) have been considered in the area of dosage form design, and the administration method has been modified. Drugs that suppress various symptoms have also been developed and used as supportive therapies to prevent AEs. Two examples of supportive therapies include antiemetics, which mitigate nausea, and granulocyte colony-stimulating factor (G-CSF), which treats neutropenia [2–4]. However, AEs are still difficult to control, and further development of supportive therapies is needed.

Cystine and theanine is a supplement that contains 700 mg cystine and 280 mg theanine; it is available in Japan and the United States. Cystine consists of two molecules of cysteine, which is a sulfur-containing amino acid, that are connected by a disulfide bond, and it is reduced and converted to cysteine in the cell. Theanine breaks down into glutamic acid and ethylamine after it is absorbed. In the cell, cysteine and glutamic acid are synthesized with glycine to form the tripeptide glutathione (GSH) [5]. GSH is reportedly the most potent antioxidant in the body, and its levels have been shown to decrease after exercise and surgery [6, 7]. The need for GSH is thought to increase under these conditions. We previously described that the intake of cystine and theanine for 10 days during the perioperative period led to the early resolution of high postoperative levels of interleukin (IL)-6 and C-reactive protein (CRP) and early recovery from changes in neutrophil and lymphocyte counts [8]. Cystine and theanine has also been shown to produce similar effects in mouse digestive tract surgery models and to prevent decreases in intestinal GSH; these effects were considered to be partly explained by the supply of GSH [9].

Justino et al. showed that the GSH concentrations in the epithelial cells of the gastrointestinal mucosa in mice decreased after the administration of 5-FU; they also found that administering the intestinal bacterial species *Saccharomyces boulardii* prevented the decrease in GSH and alleviated diarrhea, an AE associated with the administration of 5-FU [10].

Preliminary studies using cystine and theanine have shown that this treatment reduced the severity of stomatitis caused by various chemotherapies [11], suggesting that cystine and theanine is a promising supportive therapy. Therefore, we performed a prospective randomized trial in patients undergoing surgery for either colon cancer or gastric cancer with postoperative S-1 adjuvant chemotherapy to determine the preventive effects of cystine and theanine against AEs caused by chemotherapy and to evaluate the usefulness of cystine and theanine as a supportive therapy.

## Patients and methods

### Patients

This study was approved by the Institutional Review Board of Sendai City Medical Center (approval number: 2012-0010), and consent was obtained from each patient after the study was sufficiently explained. The study was performed in accordance with the Declaration of Helsinki. The subjects comprised patients who underwent R0 surgery for either colon or gastric cancer at the surgery department of the Sendai City Medical Center and who were expected to receive postoperative S-1 adjuvant chemotherapy for 4 weeks with a 2-week drug-free interval. Patients were enrolled in the study if they were in PS0 or PS1, were 20 years of age or above, submitted written consent, and fulfilled the following criteria on their pre-registration laboratory tests: white blood cell (WBC) count >3,000/mm^3^, neutrophil count >1,500/mm^3^, platelet count >100,000/mm^3^, hemoglobin level >9.0 g/dl, total bilirubin level <2.0 mg/dl, aspartate aminotransferase (AST) level <100 IU, alanine aminotransferase (ALT) level <100 IU, and estimated glomerular filtration rate (eGFR) >60 ml/min. The patient registration period lasted from July 2012 to June 2014, and the intended number of enrolled subjects was 70.

### Study design and data collection

**Fig. 1:**
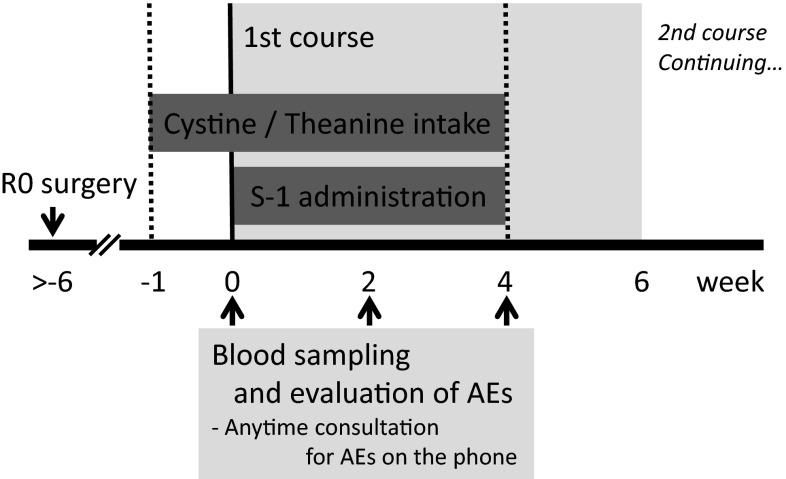
Administration schedules of S-1 and cystine and theanine after R0 surgery

The study design was a prospective randomized trial, and the subjects were allocated using the envelope method. The administration of S-1 (Taiho Pharmaceutical, Tokyo, Japan) was scheduled to be initiated within 6 weeks after surgery. The subjects in the C/T group were orally administered an amino acid supplement, which contained 700 mg cystine and 280 mg theanine (total weight, 1.7 g; Ajinomoto, Tokyo, Japan), once a day starting 1 week before and ending at the same time as the administration of S-1 (a total of 35 days) (Fig. 1). The control group received S-1 without cystine and theanine. The subjects in both groups were examined at the outpatient clinic immediately before starting S-1 chemotherapy. They underwent blood sampling, and it was confirmed that they fulfilled the inclusion criteria. The subjects visited the hospital 14 days after starting S-1 chemotherapy and at the end of the first treatment course (28 days after starting S-1 chemotherapy); during the visits, they underwent blood sampling and answered questions about AEs. The subjects were observed until the end of the first treatment course. A physician in charge of the outpatient clinic graded the AEs according to the Common Terminology Criteria for Adverse Events (CTCAE) version 4.0. If AEs that were of a grade 2 severity or more severe occurred during the chemotherapy, the administration of S-1 was suspended or discontinued, and the period in which S-1 was administered at the prescribed dose (i.e., not including the period when the dose was reduced) was recorded. Consultations regarding AEs were given by telephone and during hospital visits, and the administration of S-1 was immediately discontinued if the AE was judged to be of a grade 2 severity or more severe. The evaluation items included the incidence of AEs, which was determined through blood tests and medical interviews, and the completion rate of the first course of chemotherapy at the prescribed dose.

### Statistical analyses

The hypothesis was that the completion rate of the first course of chemotherapy would be increased from 45 % (less than half) to 80 % by administration of cystine and theanine. It was calculated that 66 samples would be required. Expecting the loss of several samples, 70 samples were included in this study.

All data were expressed as a percentage or the mean ± standard deviation (SD). The incidence of AEs and completion rate of chemotherapy were evaluated using Fisher’s exact test. The number of days that the prescribed dose of S-1 was administered in each group was compared using the *t* test. All statistical procedures were performed at a significance level of *p* < 0.05 using the Prism software package (GraphPad Software, La Jolla, CA, USA).

## Results

**Fig. 2:**
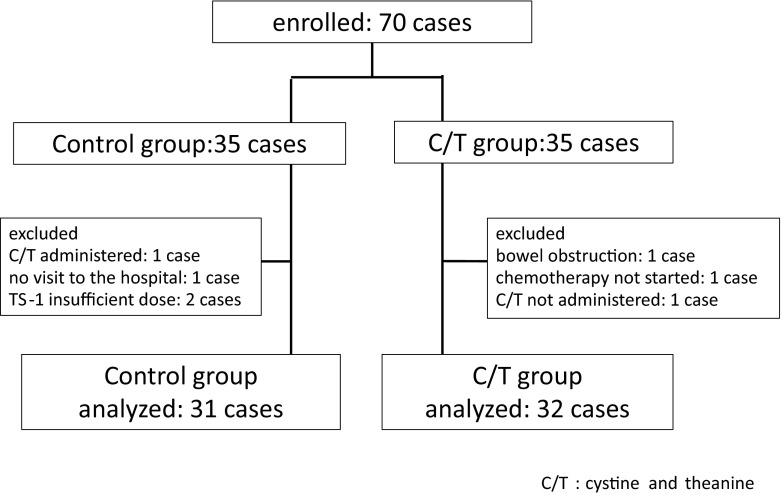
Thirty-five subjects each were randomly allocated to the control and C/T groups. After exclusion of 4 control group subjects and 3 C/T group subjects, the data from 31 and 32 subjects in the respective groups were analyzed

Of the 70 subjects who participated, 35 subjects were allocated to each group. Four subjects in the control group were excluded (1 had difficulty visiting the hospital because of low back pain, 2 were underdosed with S-1, and 1 was administered cystine and theanine), and 3 in the C/T group were excluded (1 developed ileus, 1 could not be administered S-1, and 1 was not administered cystine and theanine). Thus, 31 subjects in the control group and 32 subjects in the C/T group were evaluated (Fig. 2).

The subjects in the control and C/T groups had the following characteristics: their ages were 63.5 ± 8.9 and 63.2 ± 8.5 years, their male:female ratios were 17:14 and 21:11, and their diagnoses were colon cancer (*n* = 24 and *n* = 22) and gastric cancer (*n* = 7 and *n* = 9), respectively; 1 patient in the C/T group had gastric cancer and colon cancer (Table 1).

**Table 1:** Characteristics of the subjects in the control and C/T groups

	Control	C/T	*p* value
Age (years)	63.5 ± 8.9	63.2 ± 8.5	0.917
Sex, male:female (cases)	17:14	21:11	0.556
Colon cancer (cases)	24	22	0.517
Gastric cancer (cases)	7	9	
Gastric/colon cancer (cases)	0	1	
Total (cases)	31	32	

Age was expressed as mean ± SD and compared using the *t* test. Numbers of subjects were compared using percentage. *p* values were calculated using Fisher’s exact test

*C/T* cystine and theanine

Operative procedures in each group are shown in Table 2. Open or laparoscopic procedure was not distinguished.

**Table 2:** Operative procedures in the control and C/T groups

		Control (cases)	C/T (cases)	Total (cases)
Colon cancer	Rt. colectomy	5	8	13
	Sigmoid colectomy	8	5	13
	Resection of rectum	6	6	12
	Miles’ operation	5	2	7
	Partial resection	0	1	1
Gastric cancer	Distal gastrectomy	5	3	8
	Total gastrectomy	2	6	8
Gastric/colon cancer	Distal gastrectomy + rt. colectomy	0	1	1
Total (cases)		31	32	63

*C/T* cystine and theanine

The following incidences for AEs (any grades) were observed in the control and C/T groups, respectively: 16.1 % and 9.4 % for neutropenia, 29.0 % and 18.8 % for stomatitis, 38.7 % and 18.8 % for appetite loss, 16.1 % and 18.8 % for nausea, 41.9 % and 9.4 % for diarrhea, 22.6 % and 9.4 % for fatigue, 32.3 % and 25.0 % for pigmentation, 6.5 % and 0 % for exanthema, 9.7 % and 0 % for fever (not accompanied by neutropenia), 9.7 % and 0 % for abdominal pain, and 3.2 % and 0 % for vertigo. The incidences for all AEs, except nausea, were lower in the C/T group than in the control group, and a significant difference (*p* < 0.01) was observed in the incidence of diarrhea (Table 3). The following incidences for AEs that were of a grade 2 or more severity (at which point S-1 administration was suspended or discontinued) were observed in the control and the C/T groups, respectively: 16.1 % and 9.4 % for neutropenia, 12.9 % and 3.1 % for stomatitis, 19.4 % and 6.3 % for appetite loss, 9.7 % and 3.1 % for nausea, 25.8 % and 3.1 % for diarrhea, 12.9 % and 0 % for fatigue, and 0 % and 0 % for pigmentation. The incidences for all these AEs were lower in the CT group than in the control group, and a significant difference (*p* < 0.05) was observed in the incidence of diarrhea. Exanthema (3.2 %), fever (9.7 %), abdominal pain (9.7 %), and vertigo (3.2 %) were noted in only the control group (Table 3). With regard to operative procedure, incidences of all AEs, except neutropenia and appetite loss in gastric cancer patients, were lower in the C/T group than in the control group in both colon cancer and gastric cancer patients. The incidence of appetite loss and diarrhea of any grade was significantly lower (*p* < 0.05 and *p* < 0.01, respectively) in the C/T group than in the control group in colon cancer patients.

**Table 3:** Incidence of adverse events (AEs) during the first course (28 days) of S-1 therapy

Group	Colon cancer		Gastric cancer		Total	
	Control (*n* = 24)	C/T (*n* = 22)	Control (*n* = 7)	C/T (*n* = 9)	Control (*n* = 31)	C/T (*n* = 32)
Adverse event (AE) (grade)	Any (G ≥ 2)	Any (G ≥ 2)	Any (G ≥ 2)	Any (G ≥ 2)	Any (G ≥ 2)	Any (G ≥ 2)
Neutropenia %	16.7 (16.7)	0 (0)	14.2 (14.2)	33.3 (33.3)	16.1 (16.1)	9.4 (9.4)
Stomatitis	29.2 (8.3)	13.6 (0)	28.6 (28.6)	22.0 (0)	29.0 (12.9)	18.8 (3.1)
Appetite loss	41.7 (20.8)	9.1* (0)	28.6 (14.2)	44.4 (22.2)	38.7 (19.4)	18.8 (6.3)
Nausea	16.7 (12.5)	18.2 (4.5)	14.2 (0)	22.2 (0)	16.1 (9.7)	18.8 (3.1)
Diarrhea	41.7 (25.0)	4.5** (4.5)	42.9 (28.6)	11.1 (0)	41.9 (25.8)	9.4** (3.1*)
Fatigue	16.7 (12.5)	9.1 (0)	42.9 (14.2)	11.1 (0)	22.6 (12.9)	9.4 (0)
Pigmentation	25.0 (0)	22.7 (0)	57.1 (0)	22.2 (0)	32.3 (0)	25.0 (0)
Exantheme	8.3 (4.2)	0 (0)	0 (0)	0 (0)	6.5 (3.2)	0 (0)
Fever	4.2 (4.2)	0 (0)	28.6 (28.6)	0 (0)	9.7 (9.7)	0 (0)
Abdominal pain	8.3 (8.3)	0 (0)	14.3 (14.3)	0 (0)	9.7 (9.7)	0 (0)
Vertigo	4.2 (4.2)	0 (0)	14.3 (14.3)	0 (0)	3.2 (3.2)	0 (0)

AEs were evaluated using the CTCAE (ver. 4.0)

*C/T* cystine and theanine

* *p* < 0.05; ** *p* < 0.01 vs. control

The percent of subjects who were able to complete the first course of chemotherapy at the prescribed dose was significantly higher (*p* < 0.01) in the C/T group (75.0 %) than in the the control group (35.5 %) (Table 4). According to sub-analyses, completion rate was significantly higher (*p* < 0.01) in C/T group (90.9 %) than control group (41.7 %) in colon cancer patients, and higher, but not significant, in C/T group (44.4 %) than control group (14.3 %) in gastric cancer patients (Table 4).

**Table 4:** Completion rate and the duration of the administration period in which S-1 should be administered at the prescribed dose without suspension or discontinuation during first treatment course (28 days)

Group	Colon cancer		Gastric cancer		Total	
	Control	C/T	Control	C/T	Control	C/T
Completion rate of first course of treatment (cases)	10/24	20/22	1/7	4/9	11/31	24/32
Percent (%)	41.7	90.9**	14.3	44.4	35.5	75.0**
Duration (days)	21.0 ± 7.4	26.8 ± 3.8**	16.3 ± 8.0	21.1 ± 6.8	20.0 ± 7.7	24.8 ± 5.8**

Duration was expressed as mean ± SD and compared using the *t* test

*C/T* cystine and theanine

** p < 0.01 vs. control

The duration of the administration period in which S-1 could be administered at the prescribed dose without suspension or discontinuation during the first treatment course (scheduled to be 28 days) was significantly longer (*p* < 0.01) in the C/T group (24.8 ± 5.8 days) than in the control group (20.0 ± 7.7 days). According to sub-analyses, the duration was significantly longer (*p* < 0.01) in the C/T group (26.8 ± 3.8 days) than in the control group (21.0 ± 7.4 days) in colon cancer patients, and longer, but not significant, in the C/T group (21.1 ± 6.8 days) than in the control group (16.3 ± 8.0 days) in gastric cancer patients (Table 4).

## Discussion

The ACTS-GC study reported that the use of S-1 adjuvant chemotherapy for treating stage II/III gastric cancer increased the 5-year survival rate after surgery alone from 61.7 % to 71.7 % and improved the hazard risk by 32 % [12]. A study in Japan that assessed the regimen of orally administering uracil, tegafur, and leucovorin (UFT/LV) as an adjuvant chemotherapy after colon cancer surgery found that it was not inferior to intravenous 5-FU and levofolinate (5-Fu/LV) therapy [13]. UFT/LV is recommended in the guidelines; however, the ACTS-CC study demonstrated that S-1 is not inferior to UFT/LV [14]. The results of this study demonstrated that S-1 adjuvant chemotherapy was effective following surgery for gastric or colon cancer.

AEs associated with the administration of S-1 have been shown to affect the duration of the administration period; S-1 administered was continued for 12 months with suspensions and dose reductions in 65.8 % (340/517) of the patients after gastrectomy in the ACTS-GC study [12] and for 6 months in 76.5 % of the patients after large bowel resection in the ACTS-CC study [14]. As dose reductions were necessary because of the occurrence of AEs in 46.5 % (158 patients) of the 340 patients who completed the therapy in the ACTS-GC study, the percent of patients who completed the therapy without dose reductions was 35.2 % (182/517 patients) [12]. In a study by Maekawa et al., S-1 was administered for 12 months in 65 % of the 40 patients after gastrectomy, and only 23 % of the patients did not require a change in the administration schedule or a reduction in dose [15]. According to sub-analyses of the ACTS-GC study results, the outcomes were more favorable among the patients who could continue receiving treatment for 12 months and the patients who received 70 % or more of the planned dose [16]. Therefore, how well AEs can be controlled and how much of the scheduled dose can be administered within the scheduled treatment period are considered to markedly affect the therapeutic outcomes of S-1 adjuvant chemotherapy.

In the present clinical trial, cystine and theanine was shown to alleviate the AEs associated with the use of S-1. Moreover, during the first course of S-1 therapy, cystine and theanine significantly increased the number of days on which S-1 could be administered and significantly improved the completion rate of the therapy at the prescribed dose from 35.5 % to 75.0 %. Especially, the completion rate of colon cancer patients improved from 41.7 % to 90.9 %. The findings also suggest that cystine and theanine alleviates all AEs, in contrast to drugs such as antiemetics, that target particular symptoms. No study has yet demonstrated improvements in the completion rate of anticancer regimens through the use of particular supportive therapies. With respect to the specific symptoms, significant suppression was observed only for diarrhea; however, the effects on the other symptoms may also be significant in large-scale studies. The AEs associated with S-1 most frequently occur during the first few treatment courses. If cystine and theanine can improve the completion rate of the first treatment course, its suppressive effects on AEs may be sustained in subsequent courses in patients who continue to take it. In a previous study, we were able to administer the S-1 regimen as scheduled without AEs by the continuous administration of cystine and theanine in a small number of patients.

With respect to the dose, the final dosage form of the cystine and theanine amino acid supplement, which contained 700 mg cystine and 280 mg theanine, weighed 1.7 g, and it was readily ingested even by patients with gastrointestinal symptoms, which is a major advantage. The findings of all the clinical trials to date are based on the combination of 700 mg cystine and 280 mg theanine; future studies should evaluate the optimal dose. In an experiment using mice, its effects were not intensified at higher doses, suggesting that there is an optimal dose [9, 17].

Although the action mechanism of cystine and theanine has not yet been elucidated in detail, it likely involves an increase in the cellular concentration of GSH in the organs that are involved in the AEs which occur. Cystine and theanine provides cysteine and glutamic acid to cells, and in combination with glycine, they increase intracellular GSH concentrations. The use of an anticancer agent is expected to increase oxidative stress in cells and to reduce the concentration of GSH, which is a potent antioxidant. Because the administration of cystine and theanine for 5 days before laparotomy suppressed decreases in GSH in the small bowel mucosa and Peyer’s patches of mice [9], this may be the mechanism responsible for alleviation of the AEs caused by anticancer chemotherapy. A previous study involving 14 patients who had colorectal cancer and underwent adjuvant chemotherapy with 5-FU and oxaliplatin reported that the incidence of sensory neuropathy was significantly lower in 5 patients who were orally administered N-acetylcysteine, a GSH precursor, at 1200 mg than in the 9 patients who were not administered the supplement [18]. Another study found that among 52 patients who had colorectal cancer and underwent oxaliplatin-based chemotherapy, a significantly stronger neuroprotective effect was observed in the group that received intravenous GSH at 1500 mg/m^2^ before the administration of oxaliplatin [19]. The regimens evaluated in these clinical studies also aimed to alleviate the AEs caused by chemotherapy by increasing the GSH concentration in normal cells.

In conclusion, the intake of the amino acids cystine and theanine at 700 and 280 mg, respectively, alleviated the AEs caused by the anticancer drug S-1, reduced the frequency of therapy suspension or discontinuation caused by AEs, and improved the completion rate of the first course of therapy.
